# Detection and Identification of Anti-Neutrophil Antibodies in Immune Neutropenia: Integrating Serology, Genotyping and Clinical Interpretation

**DOI:** 10.3390/antib15040054

**Published:** 2026-06-25

**Authors:** Elyse Moritz, Renato Cerqueira, Juliana Oliveira Martins, José O. Bordin

**Affiliations:** 1 Department of Clinical and Experimental Oncology, Division of Hematology and Hemotherapy, Universidade Federal de São Paulo, São Paulo 04037-002, Brazil; elyse.moritz@unifesp.br (E.M.); juliana.martins07@hotmail.com (J.O.M.); 2 School of Health Sciences, Universidade Municipal de São Caetano do Sul (USCS), São Caetano do Sul 09521-160, Brazil

**Keywords:** immune neutropenia, autoimmune neutropenia, granulocyte/neutrophil antibodies, granulocyte/neutrophil antigens, HNA system, granulocyte serology

## Abstract

Immune-mediated neutropenias comprise a heterogeneous group of disorders characterized by antibody-mediated destruction of neutrophils, in which the detection of anti-neutrophil antibodies remains a significant diagnostic challenge. Human neutrophil antigens (HNAs) are key targets in both autoimmune and alloimmune conditions, and their identification requires an integrated laboratory approach combining serological assays, HNA genotyping, and clinical evaluation. However, variability in assay sensitivity, the presence of low-titer or conformationally dependent antibodies, and interference from anti-HLA antibodies may lead to inconclusive or misleading results. This review summarizes the immunological mechanisms underlying anti-HNA antibody-mediated neutropenia and critically evaluates current laboratory methods, including cell-based and bead-based assays. The role of HNA genotyping in supporting antibody identification and improving diagnostic accuracy is also discussed. In addition, we highlight the importance of interpreting serological findings according to antibody specificity and clinical context. An integrated and multidisciplinary diagnostic approach is essential to ensure accurate diagnosis and appropriate clinical management, while emerging technologies may further improve antibody detection in the future.

## 1. Introduction

Immune neutropenia represents a common cause of chronic neutropenia in both pediatric and adult populations, characterized by a reduction in the absolute neutrophil count (ANC) relative to age-specific reference values. As the “linchpin” of the innate immune system, neutrophils constitute the first line of defense against pathogens through mechanisms such as phagocytosis and the release of neutrophil extracellular traps (NETs). Consequently, chronic neutropenia substantially increases the risk of recurrent infections. While its etiology spans from congenital marrow failure to drug-induced toxicity, immune-mediated mechanisms involving antibodies against human neutrophil antigens (HNA) are central to many cases [1].

Following a brief overview of the etiology of chronic neutropenia and the main characteristics of HNAs, this review provides a comprehensive analysis of laboratory diagnostic approaches for detecting anti-HNA antibodies, with particular emphasis on technical challenges, methodological limitations, and clinical interpretation of laboratory results.

Clinical significance: Accurate identification of anti-HNA antibodies is essential for distinguishing autoimmune from alloimmune neutropenia, guiding treatment decisions, and predicting recurrence risk in subsequent pregnancies.

## 2. Immune-Mediated Neutropenias and Human Neutrophil Antigens

Chronic neutropenia is defined as an ANC below 1.5 × 10^9^/L persisting for more than three months. In infants younger than one year of age, a lower threshold is accepted, with neutropenia defined as ANC below 1.0 × 10^9^/L [1].

The etiology of chronic neutropenia is multifactorial, encompassing a wide range of conditions from autoimmune disorders to inherited genetic abnormalities. Etiologies are broadly classified into congenital and acquired forms, with immune-mediated causes representing the most common group among acquired neutropenias, particularly in childhood.

## 3. Immune Neutropenia

Immune forms of neutropenia result primarily from increased peripheral destruction of neutrophils, leading to reduced circulating cell counts. These can be broadly categorized into alloimmune and autoimmune neutropenia—providing an important framework for both clinical and laboratory investigation.

### 3.1. Neonatal Alloimmune Neutropenia (NAIN)

Neonatal alloimmune neutropenia (NAIN) affects approximately 1 in 1250 newborns and results from maternal sensitization to paternally inherited HNAs expressed on fetal neutrophils [2,3]. A reduced ANC is typically present at birth, may further decline during the first week of life, and can persist for up to six months [4]. Clinical manifestations are variable: some infants remain asymptomatic, whereas others develop omphalitis, skin infections, and fever; in rare cases, more severe complications may occur [5].

Although mechanistically distinct from autoimmune forms, NAIN provides a clear conceptual framework demonstrating that humoral targeting of neutrophil antigens can result in clinically significant neutropenia.

### 3.2. Autoimmune Neutropenia (AIN)

Autoimmune neutropenia (AIN) is classified into primary and secondary forms. Primary autoimmune neutropenia (pAIN) comprises distinct clinical phenotypes, including the classic form, long-lasting AIN, and late-onset AIN. The classic form predominantly affects young children, with an estimated annual incidence of approximately 1 per 100,000 [6]. It is characterized by early onset (typically before three years of age) and a self-limited course, with spontaneous resolution in up to 90% of cases within 36 months of diagnosis [7]. Clinically, pAIN is usually associated with mild infections and occurs in the absence of underlying systemic autoimmune disease. The condition is mediated by autoantibodies directed against HNA, most commonly those of the HNA-1 system [8,9].

The non-classical variants differ not only in age at onset and disease duration, but also in neutropenia severity, lymphocyte profiles, the frequency of associated autoimmune manifestations, and the need for granulocyte colony-stimulating factor [10]. Together, these phenotypic differences support the concept that pAIN represents a spectrum of disease rather than a single uniform entity.

Different from primary forms, secondary autoimmune neutropenia (sAIN) may occur at any age and is frequently associated with a wide range of underlying conditions, including autoimmune diseases, infections, solid organ or hematopoietic stem cell transplantation, and malignancies [6,11]. Its incidence and clinical course largely depend on the underlying disorder.

The pathogenesis of sAIN is heterogeneous and may involve antineutrophil antibodies directed against HNAs, as well as other autoantibodies, such as anti-Ro/SSA [9]. Neutropenia in this context may arise through distinct and potentially overlapping mechanisms. In addition to antibody-mediated peripheral destruction, other pathways include the effects of proinflammatory cytokines and the direct cytotoxic activity of activated T lymphocytes [6,11].

Despite the central role of anti-HNA antibodies in autoimmune neutropenia, their detection and the interpretation of test results remain technically challenging and are not consistently achieved in clinical practice. Variability in assay sensitivity, the presence of low-affinity or low-titer antibodies, and the complex and heterogeneous expression of neutrophil antigens contribute to diagnostic uncertainty.

Key diagnostic challenge: Variability in assay sensitivity, low-titer antibodies, and heterogeneous neutrophil antigen expression contribute to diagnostic uncertainty.

## 4. Human Neutrophil Antigens (HNAs)

HNAs are genetically determined, polymorphic, and immunogenic molecules expressed on the surface of neutrophils, playing a crucial role in both alloimmune and autoimmune responses. Antibodies directed against HNAs have been implicated in a variety of clinical conditions, including NAIN, primary and secondary AIN, transfusion-related acute lung injury (TRALI), febrile transfusion reactions, refractoriness to granulocyte transfusions, and kidney transplant rejection [12,13,14].

To date, fourteen HNA alleles have been officially assigned to five antigen systems by the International Society of Blood Transfusion (ISBT) (Table 1) [15,16]. The HNA-1, HNA-2, and HNA-3 systems are considered the most clinically relevant in the context of AIN and NAIN [2,7,9,13].

HNA-1 antigens are expressed on the low-affinity Fc receptor IIIb (FcγRIIIb/CD16), with approximately 1–2 × 10^5^ copies per cell. FcγRIIIb plays a role in immune complex clearance, act as a pro-adhesive molecule, and function as an activating receptor capable of triggering cellular responses, including NET formation. Individuals may carry between zero and four *FCGR3B* alleles, corresponding to the epitopes HNA-1a, -1b, -1c, and -1d [13,17]. Individuals with the HNA-1-null phenotype, who lack *FCGR3B* gene on both chromosomes, do not exhibit an increased susceptibility to autoimmune or immune complex-mediated diseases, nor an increased risk of infections [18]. However, they are prone to developing isoantibodies upon exposure to foreign HNA-1 antigens.

The high membrane density of this carrier glycoprotein makes the HNA-1 system a prominent target for both allo- and autoantibodies, with significant clinical implications. HNA-1 alloantibodies have been implicated in NAIN and TRALI [2,3,19]. HNA-1 autoantibodies—particularly anti-HNA-1a—represent the predominant specificity detected in pAIN [7,8], while a pan reactivity (pan-FcγRIIIb) is observed in sAIN [9,20].

HNA-2 is expressed on the glycosylphosphatidylinositol (GPI)-anchored glycoprotein CD177 and is distinguished by its bimodal expression pattern: in approximately 95–97% of individuals, neutrophils are divided into two subpopulations—one expressing CD177 and another lacking expression entirely. Despite the numerous single nucleotide polymorphisms (SNPs) identified within the *CD177* gene, HNA-2 antibodies do not discriminate between different variants. Consequently, antibodies produced by HNA-2-null individuals are classified as isoantibodies and have been implicated in immune-mediated neutropenia and TRALI [12,13].

Several mechanisms have been proposed to explain the HNA-2-null phenotype, including incorrect splicing, SNPs, insertions, and deletions leading to premature stop codons; however, no definitive cause has been established to date. In addition, CD177 expression can be significantly upregulated upon neutrophil activation, particularly in severe bacterial infections, pregnancy, inflammatory states, and malignancy [21].

HNA-3 is a biallelic antigen system expressed on the choline transporter-like protein 2 (CTL2). The HNA-3a and HNA-3b antigens differ by an *SLC44A2* c.455G > A substitution (rs2288094) resulting in an Arg152Gln amino acid change [16]. An additional *SLC44A2* c.451C > T substitution (rs147820753), which affects the amino acid adjacent to residue 152 and results in a Leu151Phe change, has been shown to impair the HNA-3a epitope. This alteration can reduce or even abolish neutrophil agglutination induced by certain HNA-3a-specific alloantibodies [22].

Anti-HNA-3 antibodies have recently emerged as potential contributors to autoimmune neutropenia [9]. Among these, anti-HNA-3a antibodies are of particular clinical significance due to their strong neutrophil-agglutinating capacity and their well-established association with severe and frequently fatal cases of transfusion-related acute lung injury (TRALI) [12]. Anti-HNA-3b antibodies have been implicated in other immune-mediated conditions, including kidney transplant rejection [14] and NAIN [23].

## 5. Mechanisms of Neutrophil Destruction in Immune Neutropenia

Anti-HNA antibodies promote neutrophil destruction through multiple, often synergistic, immune effector mechanisms. Upon binding to specific antigens on the neutrophil surface, these antibodies mediate opsonization, marking cells for recognition and removal predominantly by the reticuloendothelial system, particularly in the spleen. This process is closely linked to Fc receptor-mediated phagocytosis, in which FcγRs on macrophages and monocytes recognize IgG-coated neutrophils, leading to engulfment and intracellular degradation [24,25].

Antibody binding may also activate the complement system via the classical pathway, resulting in deposition of complement components such as C3b on the neutrophil surface, further enhancing opsonization. In some cases, terminal complement activation leads to formation of the membrane attack complex (MAC), causing direct cell lysis. Additionally, anti-HNA-1 antibodies targeting FcγRIIIb can directly activate neutrophils, triggering intracellular signaling pathways that culminate in NADPH oxidase activation, reactive oxygen species generation, and NETosis [26,27,28].

The relative contribution of these destruction pathways depends on antibody specificity, affinity, and antigen density—ultimately influencing the severity and clinical presentation of immune-mediated neutropenia.

## 6. Laboratory Methods for Anti-HNA Antibody Detection

Detection of anti-HNA antibodies relies on labor-intensive methodologies that require HNA-typed donors, specialized serological and genotyping facilities, and experienced personnel. The ISBT Granulocyte Immunobiology Working Party (GIWP) recommends a combination of techniques as a screening strategy, given that no single assay can detect all clinically relevant antibodies [29].

## 7. Granulocyte Isolation and Donor Selection

In techniques employing fresh cells—such as Granulocyte Agglutination Test (GAT), Granulocyte Immunofluorescence Test (GIFT), and Monoclonal Antibody Immobilization of Granulocyte Antigens (MAIGA)—the initial step involves isolation of neutrophils from donors with known phenotypes using dextran sedimentation followed by Ficoll density gradient separation or immunomagnetic negative selection [2,30]. Given the short lifespan of neutrophils in circulation (6–12 h), the entire process from blood collection to antibody detection must be completed on the same day. This is particularly critical for GAT, in which cells cannot be fixed with paraformaldehyde (PFA) [31].

The granulocyte panel typically consists of cells from at least three donors selected according to their HNA genotypes and expanded when necessary [29,32]. In GAT and GIFT, positive results may also provide indirect information regarding antibody specificity based on the HNA phenotypes of the selected donor panel.

## 8. Granulocyte Agglutination Test (GAT)

The GAT is based on the intrinsic ability of granulocytes to extend pseudopods, migrate toward one another, and aggregate in the presence of activating antibodies. Due to the strong agglutinating capacity of anti-HNA-3a antibodies, GAT is considered the method of choice for their detection. However, antibodies against HNA-1a, -1b, -1c, HNA-2, HNA-3b, and anti-HLA-A2 can also be effectively detected [31,33]. The assay involves incubating patient serum with a granulocyte panel and assessing the formation of cell aggregates resulting from antigen–antibody interactions. Critically, granulocytes used in this assay must not be fixed with paraformaldehyde (PFA), as fixation abolishes the functional response required for agglutination. Technical details have been described previously [2,32].

## 9. Granulocyte Immunofluorescence Test (GIFT)

GIFT is based on incubation of PFA-fixed or unfixed granulocytes with test serum, followed by staining with a fluorochrome-conjugated anti-human IgG secondary antibody and analysis by flow cytometry or fluorescence microscopy. GIFT is considered a sensitive method for detection of most HNA auto- and alloantibodies; however, antibodies against HNA-3 may yield weak or negative results. The use of HNA-transfected cell lines (e.g., HEK293) may circumvent the need for fresh granulocyte isolation, although these remain available only in a limited number of specialized laboratories [34,35]. Technical aspects of the assay have been described elsewhere [2,32].

## 10. Monoclonal Antibody-Specific Immobilization of Granulocyte Antigens (MAIGA)

MAIGA is a sandwich ELISA designed to identify HNA antibody specificities, particularly following a positive GIFT and/or GAT screening, even in the presence of concomitant HLA antibodies [33]. The assay is based on the recognition of antigen–antibody complexes by monoclonal antibodies directed against specific HNA or HLA carrier glycoproteins, which are subsequently captured on microplates coated with anti-mouse IgG. Detection is achieved using an enzyme-conjugated anti-human IgG antibody, enabling precise characterization of anti-HNA specificity, which depends on the monoclonal antibody selected for the assay [32,36].

A major advantage of MAIGA—as well as GAT and GIFT—is that serum antibodies bind to native glycoproteins on the neutrophil surface, thereby preserving epitope conformation. This is particularly important for the CTL2 glycoprotein, which carries the HNA-3a and HNA-3b epitopes. Importantly, MAIGA can now be applied to detect antibodies against all five HNA systems, following the recent availability of commercial CTL2-specific monoclonal antibody.

## 11. Bead-Based Assays

In bead-based assays, fresh cells are replaced by fluorescent microbeads coated with recombinant peptides containing HNA epitopes—eliminating the need for donor-derived cells and enabling commercialization and automation. The commercially available LABScreen Multi assay (One Lambda/Luminex) detects anti-HNA antibodies while simultaneously screening for anti-HLA class I and class II antibodies. While this approach performs well for HNA-1 and HNA-2 systems, its performance may be reduced for antibodies targeting conformational epitopes (e.g., HNA-3), with approximately 10% of anti-HNA-3 antibodies remaining undetected [37]. Conversely, bead-based assays may demonstrate higher sensitivity than conventional cell-based techniques, particularly for the detection of low-titer antibodies. Notably, in our cohort of 15 AIN cases, autoantibodies were identified exclusively by the bead-based assay in 5 of 15 patients (33.3%) [9]—underscoring the clinical value of this complementary approach.

Antibodies against the HNA-4 and HNA-5 systems may also be influenced by the loss of native antigen conformation; however, the limited availability of well-characterized sera with these specificities has hindered comprehensive validation.

**Figure 1:**
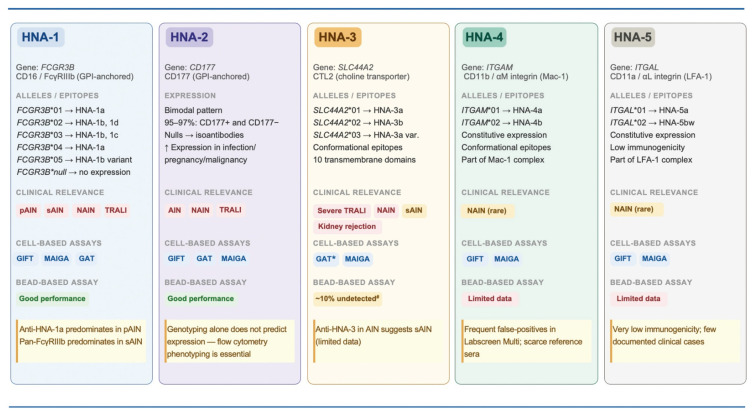
Overview of the human neutrophil antigen (HNA) systems: molecular characteristics, clinical relevance, and performance of antibody detection methods according to HNA specificity. * GAT remains the preferred method for the detection of anti-HNA-3 antibodies according to current literature. ^#^ Approximately 10% of anti-HNA-3 antibodies detected by GAT were not identified by LABScreen Multi [37]. Abbreviations: pAIN, primary autoimmune neutropenia; sAIN, secondary autoimmune neutropenia; NAIN, neonatal alloimmune neutropenia; TRALI, transfusion-related acute lung injury; GAT, granulocyte agglutination test; GIFT, granulocyte immunofluorescence test; MAIGA, monoclonal antibody-specific immobilization of granulocyte antigens; ↑ indicates an increase.

Overall, this technology represents a promising approach that enables high-throughput testing; however, it should still be used in combination with cell-based assays such as GIFT and GAT, with additional confirmation by MAIGA when necessary. The combined use of complementary techniques remains the gold standard in granulocyte serology [29]. An overview of the HNA systems and current antibody detection methods is presented in Figure 1.

## 12. HNA Genotyping

HNA genotyping has become an essential component in the diagnostic work-up of immune-mediated neutropenias, complementing serological assays and significantly enhancing the accuracy of antibody identification and clinical interpretation.

One primary role of HNA genotyping is to support the identification and confirmation of antibody specificity. By determining the patient’s HNA genotype, it becomes possible to assess whether an antibody is directed against a missing antigen (suggesting alloimmunization) or against self-antigens (supporting an autoimmune mechanism)—a distinction critical for both diagnosis and clinical management [25,31].

In NAIN, HNA genotyping is essential for establishing maternal–fetal incompatibility. Genotyping of both parents and the neonate allows: (1) identification of the incompatible antigen; (2) confirmation of the alloimmune nature of the disease; and (3) risk assessment for future pregnancies [12,25].

Another key application of HNA genotyping is the optimization of donor panels used in serological assays. The reliability of techniques such as GIFT, GAT, and MAIGA is highly dependent on the antigenic composition of the donor neutrophils included in the panel. This is particularly important for accurately detecting antibodies against polymorphic systems such as HNA-1 and for avoiding false-negative results due to lack of appropriate antigen targets in the panel [31,32].

In addition, knowledge of donor zygosity (homozygous vs. heterozygous expression) may influence antigen density and, consequently, antibody binding strength, thereby impacting assay sensitivity. Moreover, genotyping can guide repeat testing strategies by identifying the most informative donor cells to be used in subsequent assays [2,8].

PCR with sequence-specific primers (PCR-SSP) is the most commonly used method and can be combined with PCR with restriction fragment length polymorphism (PCR-RFLP) for the HNA-3 system to avoid interference from adjacent variants. Cost-effective, high-throughput TaqMan assays have been developed for HNA-1, HNA-3, HNA-4, and HNA-5 genotyping. More labor-intensive sequence-based typing methods may be used to distinguish alleles encoding HNA-1a, HNA-1b, HNA-1c, and HNA-1d [3,32,38].

An important caveat pertains to the HNA-2 (CD177) system: genotyping alone does not reliably predict antigen expression due to the presence of a pseudogene and complex regulation at transcriptional and post-transcriptional levels [39]. In this context, phenotyping by flow cytometry using monoclonal antibodies is the most appropriate strategy for HNA-2 characterization.

## 13. Biological, Technical and Clinical Challenges in Diagnosis

The identification of neutrophil-directed antibodies is a key component in elucidating the mechanisms underlying immune-mediated neutropenia. However, granulocyte immunobiology diagnostics remain restricted to a limited number of specialized laboratories worldwide. A recent international survey identified only 17 well-established laboratories performing HNA typing and serological testing globally [40].

The detection and the interpretation of test results remain challenging and are not consistently achieved in clinical practice. Variability in assay sensitivity, the presence of low-affinity or low-titer antibodies, and the complex, heterogeneous expression of neutrophil antigens all contribute to diagnostic uncertainty. No single assay is capable of detecting all clinically relevant antibodies; therefore, combined use of complementary methodologies is essential. Although the combination of GAT and GIFT is recommended as a primary screening strategy by the ISBT GIWP [29], our experience supports the concomitant use of bead-based assays to enhance diagnostic sensitivity [9].

Result discordance—whether between different assay platforms or upon repeat testing with the same method—is not uncommon in granulocyte serology and represents a meaningful diagnostic challenge. Discordance may arise from differences in antigen presentation (native vs. recombinant), antibody affinity, and the dynamic nature of antigen expression on donor neutrophils. Low-titer autoantibodies are particularly prone to inconsistent results: they may be detected exclusively by bead-based assays due to their higher analytical sensitivity, while remaining negative in cell-based methods such as GAT and GIFT. Conversely, conformationally dependent antibodies—such as certain anti-HNA-3 specificities—may be missed by bead-based platforms but detected by GAT. In cases of inter-assay discordance, MAIGA confirmation and repeat testing with a fresh sample are recommended before clinical conclusions are drawn.

## 14. Patient-Related Factors Affecting Assay Performance

Several patient-related factors may contribute to reduce assay sensitivity. Neutrophil activation under inflammatory conditions is associated with increased plasma levels of soluble FcγRIIIb (sFcγRIIIb) [41], especially in HNA-1b individuals [42]. This may promote antibody autoadsorption, reducing the availability of circulating antibodies for detection. Furthermore, inflammatory states may lead to decreased surface expression of HNA-1 antigens [43].

These biological mechanisms may also help explain the paradoxical increase in neutrophil counts observed in some patients with autoimmune neutropenia during inflammatory or infectious episodes. In addition to increased bone marrow production, enhanced antibody sequestration by soluble antigens and reduced surface antigen expression may decrease antibody-mediated clearance, rendering neutrophils less susceptible to immune destruction.

Similarly, administration of granulocyte colony-stimulating factor (G-CSF) has been shown to significantly reduce FcγRIIIb expression while increasing neutrophil activation, turnover and migration. This results in the sustained release of soluble FcγRIIIb with elevated plasma levels persisting for several days [44]. Consequently, patients receiving G-CSF may present reduced likelihood of anti-HNA-1 antibody detection—a critical consideration when scheduling serological testing.

## 15. Donor-Related Factors Affecting Assay Performance

Variations in antigen expression may occur in neutrophils obtained from donors with underlying inflammatory conditions. Reduced surface expression of HNA-1 antigens [43,45] and increased expression of HNA-2 [21] may alter antigen density and consequently affect antibody binding. This represents a potential source of variability in cell-based assays such as GAT and GIFT, which rely on freshly isolated donor neutrophils. In addition to affecting assay sensitivity, these factors may compromise standardization and contribute to both intra- and interlaboratory variability.

In this context, techniques that provide a more stable and homogeneous source of antigens, such as bead-based assays and transfected cell systems, may offer important advantages for antibody detection. Nevertheless, further studies are needed to better define the relationship between antigen expression on donor neutrophils, circulating levels of soluble HNAs in patients, and assay sensitivity, particularly under inflammatory conditions.

## 16. Anti-HLA Antibody Interference

A well-recognized limitation is the interference from anti-HLA antibodies, especially in patients with a history of pregnancy or transfusion. Since GAT and GIFT rely on donor granulocytes expressing HLA class I antigens—and, upon activation, HLA class II—positive reactions may reflect anti-HLA rather than true anti-HNA reactivity [12]. Pre-analytical absorption of HLA class I antibodies using pooled platelets represents a feasible strategy, though it does not address HLA class II antibody contribution. The combined use of MAIGA and bead-based platforms—which are not affected by anti-HLA reactivity—represents a valuable complementary strategy [2,3].

## 17. Challenges in the Detection of Anti-HNA-3

The HNA-3 system (HNA-3a and HNA-3b) is located on CTL2, a complex transmembrane protein with ten hydrophobic membrane-spanning domains, five extracellular loops, and six intracellular regions. This structural complexity poses significant challenges for recombinant antigen-based assays, as antibody recognition may depend on native conformation and post-translational modifications [34]. Schulz et al. (2017) demonstrated that approximately 90% of anti-HNA-3-positive sera are correctly identified by the newer LABScreen Multi incorporating recombinant HNA-3 peptides—indicating that cell-based GAT remains essential to ensure maximal sensitivity [37].

Nevertheless, due to the high analytical sensitivity of bead-based assays, certain antibodies—particularly those with low mean fluorescence intensity (MFI)—may be detected exclusively by this method while remaining negative in conventional cell-based assays. In our previous study, low-level autoantibodies (MFI range: 1027–1470) were detected only by LABScreen Multi [9]. These observations suggest that multiple factors influence assay performance and highlight the complementary nature of different detection methodologies.

## 18. Complex Serological Patterns

**Figure 2:**
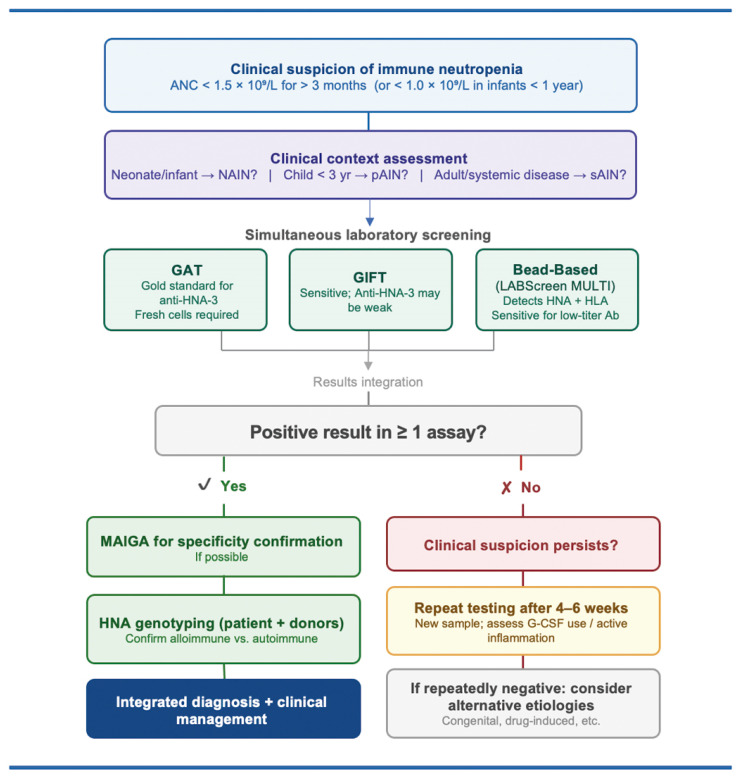
Proposed diagnostic algorithm for anti-HNA antibodies in immune neutropenia. An integrated approach of clinical evaluation, serology and genotyping. Abbreviations: ANC, absolute neutrophil count; pAIN, primary autoimmune neutropenia; sAIN, secondary autoimmune neutropenia; NAIN, neonatal alloimmune neutropenia; GAT, granulocyte agglutination test; GIFT, granulocyte immunofluorescence test; MAIGA, monoclonal antibody-specific immobilization of granulocyte antigens; G-CSF, granulocyte colony-stimulating factor.

In the evaluation of anti-neutrophil antibodies, complex serological patterns are frequently encountered. Weak or borderline reactivity in a single assay may reflect low-affinity antibodies, assay-specific sensitivity, or technical variability rather than true antigen specificity [25,31]. Conversely, negative results do not exclude immune-mediated neutropenia, particularly in cases with strong clinical suspicion [29,31]. Repeat testing with a newly collected sample after an appropriate interval is recommended, as antibody levels may fluctuate over time or become detectable during disease progression [7,25]. Based on the presented data, a comprehensive diagnostic workflow is proposed in Figure 2. This workflow reflects current practice in our institution and integrates the complementary strengths of each method. Prospective evaluation of its clinical performance—particularly the diagnostic gain achieved by incorporating bead-based assays into routine screening—would be highly relevant and could be the subject of a future comparative study.

Diagnostic principle: Negative antibody testing does not exclude immune neutropenia. Repeat testing after 4–6 weeks and cross-validation with a complementary assay platform are recommended when clinical suspicion remains high.

## 19. Clinical Interpretation According to Anti-HNA Specificity and Disease Context

Accurate clinical interpretation of anti-HNA antibody testing requires integration of serologic findings with antibody specificity, HNA genotyping, and clinical context. This comprehensive approach is essential, as anti-HNA reactivity may reflect either autoimmune or alloimmune mechanisms—each with distinct diagnostic and therapeutic implications.

In neutropenic neonates and infants up to 6 months of age, NAIN must be systematically considered [4]. Combined assessment of maternal and neonatal HNA genotypes, together with antibody specificity, is required to establish an alloimmune etiology and to avoid misclassification as autoimmune disease.

Beyond the neonatal period, alloimmunization to HNAs may also occur in transfused patients and multiparous women as a consequence of cumulative antigen exposure. Population-based studies have demonstrated considerable variability in HNA allele frequencies and incompatibility rates, supporting a measurable risk of alloantibody formation following transfusion or pregnancy [2,23,46]. These alloantibodies may interfere with the investigation of autoantibodies and complicate test interpretation, particularly in cases lacking clearly defined antibody specificity or HNA genotyping. In suspected autoimmune scenarios, detection of anti-HNA reactivity without confirmation of its immune origin may therefore be misleading. Failure to distinguish between alloimmune and autoimmune reactivity may obscure the underlying mechanism of neutropenia and delay appropriate diagnostic clarification.

In contrast to the well-defined alloimmune mechanism observed in NAIN, pAIN exhibits a relatively consistent serologic profile. Across pediatric cohorts, autoantibodies are predominantly directed against the HNA-1 and HNA-2 systems, with progressive refinement toward defined specificity over time. Although early phases of the disease may occasionally show broader reactivity, longitudinal studies demonstrate evolution toward discrete anti-HNA-1a or anti-HNA-1b specificity [8].

A distinct pattern is observed in sAIN, which occurs in association with a wide range of underlying conditions and displays the greatest degree of immunologic heterogeneity. Both early and contemporary studies indicate that non-primary forms, including the sAIN spectrum, are frequently associated with nonspecific or pan-reactive antibody patterns, without consistent refinement toward defined HNA epitopes [8,9]. Cases of simultaneous reactivity against multiple HNA specificities have been described, albeit infrequently. Bruin et al. reported a patient with primary AIN whose serum contained antibodies with concurrent NA1, pan-FcγRIIIb, and CD11a specificities, a finding associated with a prolonged disease course exceeding six years [20]. Additional weak reactivities against CD11a or CD11b were also identified by MAIGA in a subset of primary AIN sera from the same cohort [20]. In secondary AIN, the broad pan-FcγRIIIb reactivity pattern may itself reflect multiepitope targeting, and the immunological heterogeneity of this form raises the possibility of simultaneous involvement of more than one antigen system. When multiple specificities are detected, HNA genotyping becomes particularly important to determine whether each reactivity represents an autoimmune or alloimmune response, and MAIGA confirmation for each specificity is advisable when feasible. Systematic data on the frequency and clinical impact of multi-system HNA positivity remain limited and warrant further investigation. In addition, recent data have identified anti-HNA-3 autoantibodies exclusively in secondary forms, further supporting the concept that sAIN encompasses distinct immunologic mechanisms not observed in primary disease [9].

**Figure 3:**
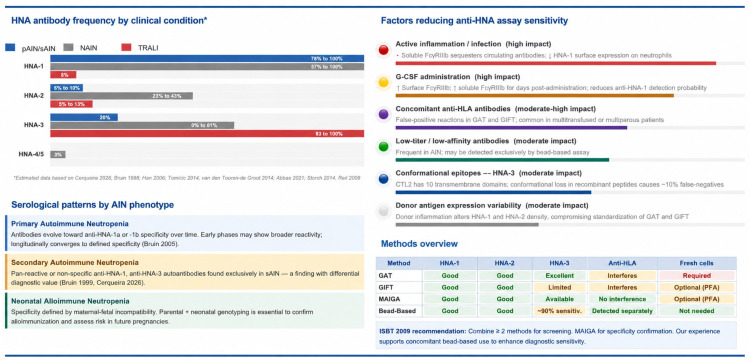
Overview of immune neutropenia and major diagnostic challenges. The figure summarizes HNA antibody specificities and their frequencies according to different clinical conditions [2,9,20,47,48,49,50,51]; characteristic serological patterns associated with distinct etiologies of neutropenia; biological and technical factors influencing assay sensitivity; and currently available laboratory methods for anti-HNA antibody detection. Abbreviations: pAIN, primary autoimmune neutropenia; sAIN, secondary autoimmune neutropenia; NAIN, neonatal alloimmune neutropenia; TRALI, transfusion-related acute lung injury; GAT, granulocyte agglutination test; GIFT, granulocyte immunofluorescence test; HNA, human neutrophil antigens; MAIGA, monoclonal antibody-specific immobilization of granulocyte antigens; G-CSF, granulocyte colony-stimulating factor; ↑ indicates an increase; ↓ indicates a decrease. Estimated data based on Bruin (1999), Han (2006), Reil (2008), Storch (2014), Tomicic (2014), van den Tooren-de Groot (2014), Abbas (2021), and Cerqueira (2026).

Taken together, these findings demonstrate that anti-HNA antibody profiles are not uniform across immune neutropenias but instead reflect the underlying immunopathogenic context. Accordingly, interpretation of serologic results should not rely solely on antibody detection, but rather on an integrated approach combining antibody specificity, HNA genotyping, and clinical features. This multidisciplinary framework is essential to distinguish between autoimmune and alloimmune neutropenia and to ensure accurate diagnosis and appropriate clinical management. Important aspects of immune neutropenia and diagnostic challenges are summarized in Figure 3.

Clinical pearls: (A) Anti-HNA-3 antibodies in AIN suggest a secondary etiology. (B) Evolving toward anti-HNA-1a or -1b specificity over time is characteristic of primary AIN. (C) Failure to distinguish alloimmune from autoimmune reactivity may delay appropriate diagnosis and management.

## 20. Conclusions

The detection and interpretation of anti-neutrophil antibodies remain among the most complex challenges in diagnostic immunohematology. No single laboratory method is sufficient to capture the full spectrum of clinically relevant anti-HNA antibodies. Instead, accurate diagnosis relies on the integration of complementary serological techniques, HNA genotyping, and careful clinical correlation.

Anti-HNA antibodies cannot be interpreted in isolation. Their clinical significance is intrinsically linked to the immunological context in which they arise—whether autoimmune or alloimmune—and to the specific antigen systems involved. Distinct serological patterns across primary and secondary autoimmune neutropenias underscore the heterogeneity of these conditions and highlight the need for individualized diagnostic reasoning.

In parallel, both biological and technical factors—including antigen variability, inflammatory modulation of neutrophil surface expression, and assay-related limitations—significantly influence antibody detectability and must be considered when interpreting laboratory results. These challenges reinforce the critical role of specialized laboratories and emphasize the need for standardized diagnostic workflows.

Looking forward, advances in molecular typing, high-throughput platforms, and cell-based engineered systems hold promise for improving both sensitivity and specificity in anti-HNA antibody detection. However, their implementation should complement—not replace—the current multimodal approach.

Ultimately, the integration of serology, genotyping, and clinical insight represents the cornerstone of accurate diagnosis in immune neutropenias. Establishing structured diagnostic algorithms and strengthening collaboration between clinicians and specialized laboratories will be essential to translate laboratory findings into meaningful clinical decisions and to improve patient outcomes.

## Figures and Tables

**Table 1:** HNA systems, associated alleles and epitopes [16].

System	Glycoprotein	Allele	Epitope (s)
HNA-1	CD16/FcγRIIIb	CD177*01	HNA-1a
		FCGR3B*02	HNA-1b, HNA-1d
		FCGR3B*03	HNA-1b, HNA-1c
		FCGR3B*04	HNA-1a
		FCGR3B*05	HNA-1b variant
	No glycoprotein	FCGR3B*null	HNA-1 null
HNA-2	CD177	*CD177*	HNA-2
	No glycoprotein	No allele	HNA-2 null
HNA-3	CLT2/SLC44A2	SLC44A2*01	HNA-3a
		SLC44A2*02	HNA-3b
		SLC44A2*03	HNA-3a variant
HNA-4	CD11b/CD18/ITGAM	ITGAM*01	HNA-4a
		ITGAM*02	HNA-4b
HNA-5	CD11a/CD18/ITGAL	ITGAL*01	HNA-5a
		ITGAL*02	HNA-5b

## Data Availability

No new data were created or analyzed in this study. Data sharing is not applicable to this article.

## Abbreviations

The following abbreviations are used in this manuscript:

AIN	Autoimmune neutropenia
anti-HNA	Anti-human neutrophil antigen antibody
CTL2	Choline transporter-like protein 2
FcγRIIIb	Fc gamma receptor IIIb
GAT	Granulocyte agglutination test
G-CSF	Granulocyte colony-stimulating factor
GIFT	Granulocyte immunofluorescence test
GIWP	Granulocyte Immunobiology Working Party
HLA	Human leukocyte antigen
HNA	Human neutrophil antigen
ISBT	International Society of Blood Transfusion
MAIGA	Monoclonal antibody immobilization of granulocyte antigens
MFI	Mean fluorescence intensity
NAIN	Neonatal alloimmune neutropenia
NETosis	Neutrophil extracellular trap formation
pAIN	Primary autoimmune neutropenia
PCR-RFLP	Polymerase chain reaction–restriction fragment length polymorphism
PCR-SSP	Polymerase chain reaction with sequence-specific primers
PFA	Paraformaldehyde
sAIN	Secondary autoimmune neutropenia
sFcγRIIIb	Soluble Fc gamma receptor IIIb
TRALI	Transfusion-related acute lung injury
